# Terahertz microscopy through complex media

**DOI:** 10.1038/s41598-025-95951-6

**Published:** 2025-04-05

**Authors:** Vivek Kumar, Vittorio Cecconi, Antonio Cutrona, Luke Peters, Luana Olivieri, Juan S. Totero Gongora, Alessia Pasquazi, Marco Peccianti

**Affiliations:** 1 Emergent Photonics Lab (EPic), Department of Physics and Astronomy, University of Sussex, Brighton, BN1 9QH UK; 2 Emergent Photonics Research Centre, Department of Physics, School of Science, Loughborough University, Loughborough, LE11 3TU UK; 3 Laboratoire Kastler Brossel, ENS-Université PSL, CNRS, Sorbonne Université, Collège de France, 24 rue Lhomond, Paris, 75005 France

**Keywords:** Terahertz optics, Nonlinear optics, Imaging and sensing

## Abstract

Manipulating broadband fields in scattering media is a modern challenge across photonics and other wave domains. Recent studies have shown that complex propagation in scattering media can be harnessed to manipulate broadband light wave packets in space-time for focusing, imaging, and computing applications. Interestingly, while many proposed methodologies operate on intensity-based assessment of scattered fields, often in the spectral domain, from a pure transmission-function perspective, scattering operates as a linear field-level combinatory process, i.e., the superposition of transformation of unit excitations. As a result, we recently demonstrated that gaining experimental access to instantaneous scattered fields, as available through time-domain spectroscopy in the terahertz (THz) spectral range, in conjunction with sparse light excitation typical of ghost imaging, provides a key advantage in enabling the functionalisation of scattering, exposing a novel modelling paradigm. In this paper, we provide experimental proof of reconstructing 1-dimensional object features through a scattering medium using a fully broadband THz time-domain approach.

**Supplementary Information:**

The online version contains supplementary material available at 10.1038/s41598-025-95951-6.

## Introduction

Inhomogeneous complex media, such as biological tissues or atmospheric turbulence, exhibit wave scattering, typically viewed as an inevitable perturbation or a nuisance. Due to the recurrence of scattering and interference, this phenomenon seemingly obliterates both the spatial and time information of any transmitted wave^1,2^. As commonly experienced, scattering ultimately limits imaging, a challenge so fundamental that it is the sole focus of a large multi-domain research effort. However, multiple scattering is a highly complex but deterministic process; hence, information is scrambled but not completely lost. Wave propagation modelling in scattering media is an invaluable field of research and is regarded as a key objective in free-space communication and biomedical imaging^3–5^. Significant advancements have addressed wave control through disordered materials, resulting in scattering-assisted images using wavefront shaping techniques^6–9^. Within this research area, wavefront shaping methods have been explored in high-resolution imaging modalities such as optical coherence tomography^10^, fluorescence microscopy^11^, two-photon microscopy^12,13^, photoacoustic microscopy^14^ and improved resolution of images. These imaging modalities demonstrated that controlling the propagation of scattered light can be essentially addressed by optimising an input wavefront, a process that can be deployed via feedback optimisation^15,16^, phase conjugation^17,18^ or by measuring the optical transfer matrix^19–22^.

In its most basic embodiment, the deterministic approach towards space-time wave synthesis in scattering media is pivoted onto the idea of probing the media with spatially orthogonal illuminations and sensing their corresponding scattered wave at the output. For a sufficiently complex medium, this means that the spatiotemporal field pattern at the output can be associated with each input sampling function (or pattern) without ambiguity, as the transformation between input and output can be approximated as unique -although time-reversal would require the observation of the scattered wave in all directions, including the backscattering at the input. Via the application of the theoretical foundation presented in ref^23,24^, scattering can be managed as a linear combinatory process. Once a spatiotemporal output set is recorded for a series of orthogonal input illumination, we can project an arbitrary desired scattered field onto this set, determining the required input excitation. Indeed, this is not a statistically founded concept, and it is not based on any specific portion of the scattered field (i.e., ballistic or diffusing). Notably, the deterministic probing of the combinatory scattering element of a sample takes into account the surface scattering, and it is solely based on the orthogonality of the detected output pattern to reconstruct unambiguous input-output linear transformation.

Trivially, in this vision, direct access to the instantaneous scattered fields is essential from an experimental point of view, as the superposition principle does not generally apply to photonic intensity-based scattering assessments. Additionally, in state-of-the-art, the extension of this concept to broadband illumination is challenged by the fact that the combinatory scattering element is, in general, a function of the frequency, while this aspect is marginally relevant when the superposition of scattered spatiotemporal waveforms is considered. Modern THz-time-domain spectroscopy (TDS) enables a direct route to measure the electric field oscillations in broadband pulses^25^. Therefore, an intriguing question is whether working with the spatiotemporal electric field and the prospect of field-sensitive detection may shed light on reconstructing the combinatory scattering element of a sample on a broadband spectrum. In such a direction, we recently demonstrated field-level THz wave synthesis^26^.

The core research literature in scattering functionalisation in photonics has presented a significant use of off-the-shelves spatial light modulators. Interestingly, spatial light modulation is somewhat challenging in the THz domain^27^. The spatial modulation of THz waves has predominantly relied on indirect methods, including the use of spinning disk masks^28,29^, optically controlled carrier-based masking in silicon^30–33^, meta-material spatial light modulators^34–36^, spatial encoding of optical probe beams in electro-optic imaging systems^37^ as well as optical pump beams in systems using nonlinear^38–41^ or spintronic THz emitters^42^. Many of those wavefront modulation schemes are at the basis of single-pixel ghost imaging and other forms of imaging analysis. The use of THz spatial light modulation for achieving comprehensive wavefront control through scattering media remains at an embryonic stage. We can also observe that in this context, while typical illuminated areas in THz experimental embodiments recall similar implementations in optics, the spatial density of independent input modes in a scattering media is several orders of magnitude lower. This means that available diffraction-limited spatial light modulation cannot access a significant diversity of propagating modes in scattering samples^43^. As a possible solution implemented here, a near-field coupled knife-edge can be used to modulate the spatial properties of an input light field, exposing modes that are not coupled with input radiative fields. Here, we demonstrate the 1D imaging through scattering via a THz-TDS imager to decompose the time-domain scattered field at the output facet. The spatiotemporally resolved field-based measurements correspond to the set of spatially modulated THz wavefronts. The modulation set is simply obtained by scanning the input facet of the scattered 1D knife-edge placed in the near-field.

## Results and discussion

### Spatiotemporal THz field mixing in scattering media

We begin addressing how fields at source and detection planes are connected by a complex combinatory matrix element of a scatterer precisely for the case of broadband pulse illumination. We define the input-output field relationship via the space-time impulse response of scattering media, $\:{T}_{x}\left(x,y;{x}^{{\prime\:}},{y}^{{\prime\:}};t,{t}^{{\prime\:}}\right)$, as

1 $$\:{E}_{d}\left({x}^{{\prime\:}},{y}^{{\prime\:}},{z}_{d};{t}^{{\prime\:}}\right)=\iiint\:{T}_{x}\left(x,y;{x}^{{\prime\:}},{y}^{{\prime\:}};t,{t}^{{\prime\:}}\right){E}_{s}\left(x,y,{z}_{s};t\right)dxdydt$$

where, $\:{E}_{d}\left({E}_{s}\right)$ is spatiotemporal field distribution at the detection/source plane in the cartesian coordinates defined by $\:({x}^{{\prime\:}},{y}^{{\prime\:}},{z}_{d})/(x,y,{z}_{s}$), respectively. In frequency domain $\:\omega\:$, Eq. (1) reads as follows,

2 $$\:{\stackrel{\sim}{E}}_{d}\left({x}^{{\prime\:}},{y}^{{\prime\:}},{z}_{d};{\upomega\:}\right)=\iint\:{\stackrel{\sim}{T}}_{x}\left(x,y;{x}^{{\prime\:}},{y}^{{\prime\:}};{\upomega\:}\right){\stackrel{\sim}{E}}_{s}\left(x,y,{z}_{s};{\upomega\:}\right)dxdy$$

where $\:{\stackrel{\sim}{E}}_{d}$, $\:{\stackrel{\sim}{E}}_{s}$ denote the time-Fourier transform of the fields at detection, source plane and $\:{\stackrel{\sim}{T}}_{x}\left(x,y;{x}^{{\prime\:}},{y}^{{\prime\:}};{\upomega\:}\right)$ represents coherent transfer function for a scattering sample.

**Fig. 1:**
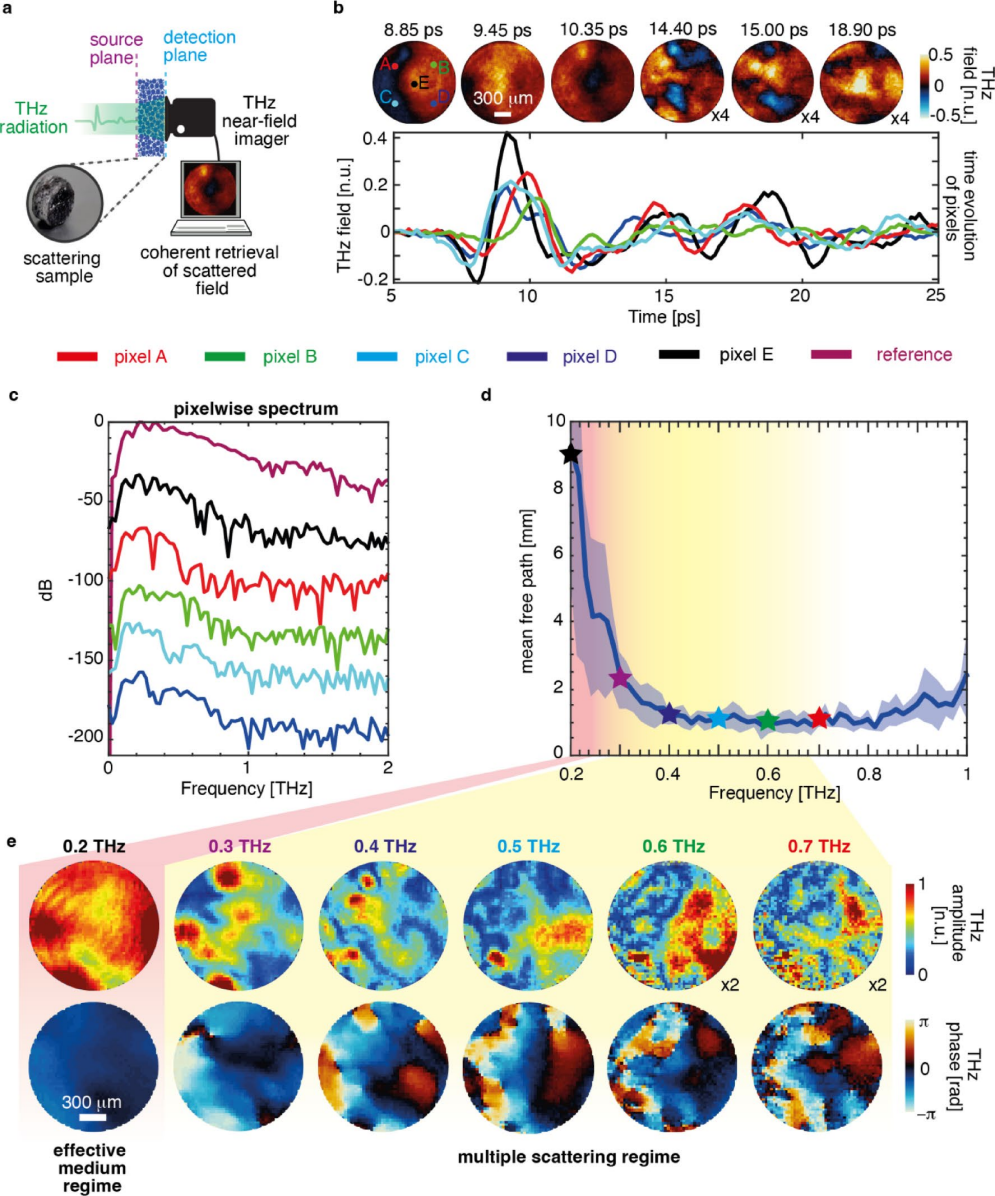
Broadband speckle formation of THz fields. (**a**) Schematic showing the projection of a pulsed THz field at the source plane of the scattering sample and the collection of the scattered field via a time-domain THz imager. (**b**) Spatiotemporal THz field transmission through scattering samples exhibits temporal distortion, as shown in the pixel-wise time evolution of the pulses and random modulation in the spatial field distributions (video 1). Field amplitude is plotted 4 times the original spatial field at $\:14.40\:\text{p}\text{s}$, $\:15.00\:\text{p}\text{s}$, and $\:18.90\:\text{p}\text{s}$. (**c**) The pixel-wise spectrum of scattered fields corresponds to the pulses shown in a. (**d**) Mean free path of the scattering sample within the THz source bandwidth (**e**) The spatial multiplexing of broadband THz fields and phases illustrates the broadband speckle synthesis within the THz source bandwidth (video 2).

THz source providing an ultrafast broadband pulse with a duration shorter than Thouless time (the typical time scale over which light diffuses across the medium ) results in randomised field transient in both space and time after propagating through scattering media^44,45^. In Fig. 1, we recorded the transmitted time-resolved THz field distribution in response to Gaussian beam illumination on the scattering sample (sample details are in supplementary S1), as shown in the schematic (Fig. 1a). As expected, scattering introduces a time-dependent morphology of the scattered field profile. Specifically, the THz field distribution resulted in complex spatial interference patterns in the transmission (Fig. 1b). The evolution of the spatial field on an ultrafast time scale shows the transition from the ballistic to the diffusive scattering regimes. Initial spatial THz field profiles at 8.85 ps and 9.45 ps show relatively more uniformity, indicating a ballistic propagation of THz waves associated with a low statistical occurrence of photon scattering, i.e., the THz field maintains its spatial momentum content. As time progresses, the diffused photons emerge, leading to an entirely randomization of the spatial field profiles due to several scattering occurrences inside the medium, shown by the spatial frames at 10.35 ps, 14.4 ps, 15 ps, and 18.9 ps. Also, the scattering process becomes more apparent in the temporal progression of distinct pixels that constitute the considerably broadened pulses in conjunction with the different time delays. However, the overall observed time delay in the pulses can be attributed to the thickness of the scattering sample (5.53 mm). As a result of the dispersive characteristics inherently present in scattering media, the temporal widening of a scattered pulse occurs concomitantly with the attenuation of its peak values. The primary cause of attenuation in the scattered pulses is attributed to the scattering of high-frequency components of the impinging pulse, as illustrated in Fig. 1c. The measured values of the mean free path ($\:{l}_{s}$) for the sample are shown in Fig. 1d. In our experiments, we observed the variation of $\:{l}_{s}\left(\omega\:\right)$ ranging between 1 mm and 10 mm within the source bandwidth. Note that because the scattering media is near-field coupled with the detector, scattering can appear highly inhomogeneous in spectral properties at the output. The spatial-spectral profiles of the THz field at 0.2–0.7 THz (Fig. 1e) exhibits the formation of broadband speckles, characterised by subwavelength spot size and disordered phase distribution. We highlight that the detection is near-field coupled with the scattering media, hence, the resolution is not bounded by the wavelength. Additionally, in our experiment, the THz source spectrum extends up to ~ 1.5 THz (supplementary figures S1 and S4), but multiple scattering attenuates higher frequencies, limiting the effective spectrum to < 1 THz (supplementary figure S1(c)); thus, analysis is performed in the 0.2–0.7 THz range where SNR is optimal and spatio-spectral characterisation is more reliable.

### Scattering media characterisation using knife-edge wavefront shaping

**Fig. 2:**
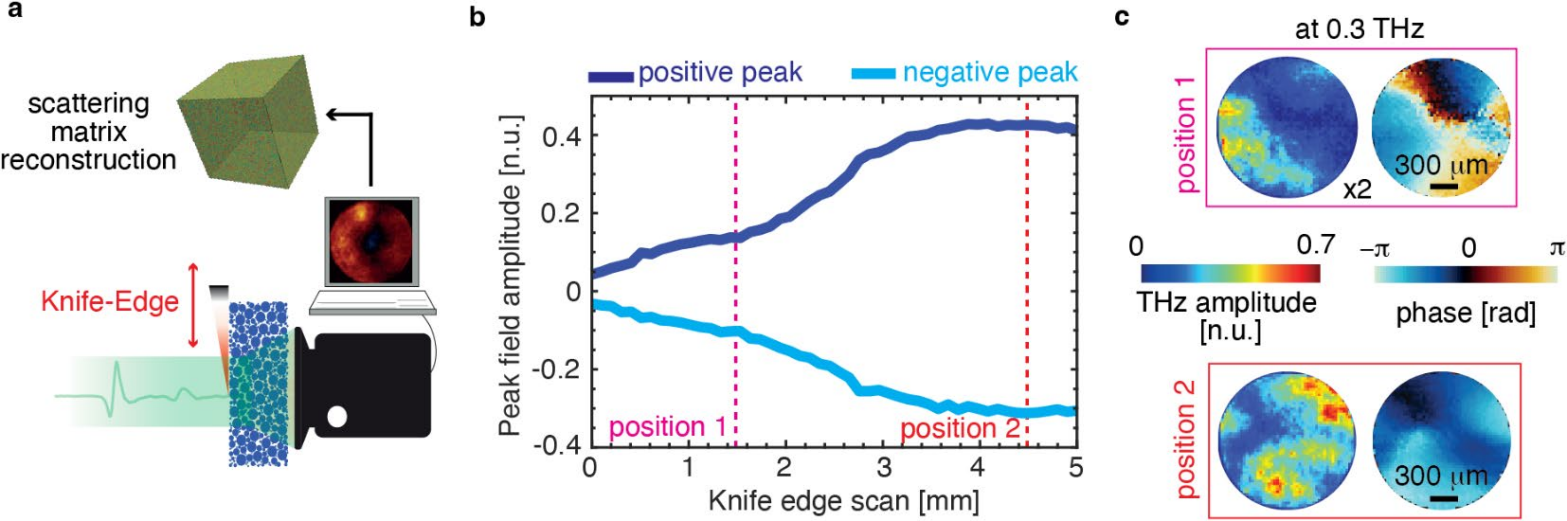
Scattering media characterisation using knife-edge wavefront shaping. (**a**) Schematics of the experimental setup showing a knife-edge shapes the impinging THz wavefront at the front end of the scattering medium (8% fractional mass concentration of Silicon microparticles embedded in paraffin) and corresponding scattered fields are collected using a THz time-domain imager placed within the near-field region. By combining the transmitted fields corresponding to the various knife-edge positions, the space of potential 1D spatiotemporal images could be derived and further constructed as a hyperspectral combinatory scattering matrix to characterise the scattering sample. (**b**) variation of pulse peak value with respect to the various transverse position of knife-edge. (**c**) spatial field amplitude and phase profile at 0.3 THz corresponding to knife-edge position 1 and 2 (dashed lines shown in **b**).

The core idea is to provide a unique scattering association to different shielding portions of the broadband THz field illumination (as per ref^46,47^) with a knife-edge moving in the near-field of the scatterer. The scatterer waveform obtained by the 2D time-domain detection set is used as a base to reconstruct a 1D transmission matrix. Once the transmission matrix is known, the image of any 1D object can be deterministically retrieved. Figure 2a illustrates a typical setting for a knife-edge (a thin conductive blade clipping a Gaussian beam) scan, where the THz near-field imager collects scattered fields from the sample partially illuminated. We can write the spatiotemporal field profile ($\:{E}_{s}$) generated by the specific placement of the knife at the source plane as,

3 $$\:{E}_{s}\left(x,y,{z}_{s};t\right)\propto\:\text{K}\left(x{,y}_{o},{z}_{s}\right){E}_{in}\left(x,y,{z}_{s};t\right)$$

where $\:\text{K}\left(x,{y}_{o},{z}_{s}\right)$ represents the point-wise spatial response of the knife-edge, which is defined as a one-dimensional Heaviside step function in the transverse direction with a fixed coordinate position $\:{(y}_{o},{z}_{s})$ and $\:{E}_{in}$ is the time-resolved Gaussian beam profile from the source. The knife-edge used in our setup is a thin conductive blade with micron-scale thickness at the sharp edge, placed approximately parallel to the scattering media input facet, and transversally translated, as shown in Fig. 2(a). Initially, we characterised the spatial beam profile for the reference THz field (included in the supplementary figure S4) to normalise the results. The key idea here is that (Fig. 2a), because of the near-field coupling, the knife-edge can selectively couple with a relatively large number of modes in the scattering volume (i.e. discernible scattering outputs) not necessarily accessible through far-field illuminations.

Figure 2b illustrates the variation in positive and negative peak values of the pulse as a function of the transverse position of the knife-edge. The scan axis is oriented to uncover more THz field as the translation increases. In the spectral domain, the spatial field amplitude and phase distribution at 0.3 THz, corresponding to the input wavefronts generated by two different knife-edge positions (at 1.5 mm and 4.5 mm), are shown in Fig. 2c, highlighting the influence of the scattered field from the knife-edge positions.

We can write fields at the source and detection plane as vectors $\:{e}_{s}\in\:{\mathbb{C}}^{S\:\times\:1}$ and $\:{e}_{d}\in\:{\mathbb{C}}^{D\:\times\:1}$, and define a scattering combinatory transfer matrix as $\:{\varvec{T}}_{ds}\in\:{\mathbb{C}}^{S\:\times\:D}$, for each frequency *ω*. S and D represent the number of independent pixels (or segments) that sample the input and output field plane, respectively. For R number of independent spectral sampling points, ***T*** is a $\:{\mathbb{C}}^{S\:\times\:D\times\:R}$ three-dimensional matrix, defined as $\:{\varvec{T}}_{ds}\left({\omega\:}_{r}\right)$. For the given r-th spectral mode, the linear relationship between generic s-th source and d-th detection spatial segments reads as follows,

4 $$\:{e}_{d}\left({\omega\:}_{r}\right)=\sum\:_{s}{\varvec{T}}_{ds}\left({\omega\:}_{r}\right){e}_{s}\left({\omega\:}_{r}\right)$$

Further, for each i-th position of the knife-edge, we collect the corresponding $\:{e}_{d}^{\left(i\right)}\left({\omega\:}_{r}\right)$ and column-wise stack in a measurement matrix $\:\varvec{M}\left({\omega\:}_{r}\right)\in\:{\mathbb{C}}^{D\:\times\:P}$ and corresponding synthesised THz wavefront in decomposition matrix $\:\varvec{X}\left({\omega\:}_{r}\right)\in\:{\mathbb{C}}^{S\:\times\:P}$, where P is the number of wavefronts modulated by the edge of the knife. To identify the transfer matrix at frequency $\:\omega\:$, we set up a least square optimisation problem as,

5 $$\:{\varvec{T}}_{r}^{\dagger}\left({\omega\:}_{r}\right)=\text{a}\text{r}\text{g}\text{m}\text{i}{\text{n}}_{\:{T}_{r}\in\:{\complement\:}^{S\:\times\:D}}\:\frac{1}{2}\:{\left\| {\varvec{M}\left({\omega\:}_{r}\right)}^{\dagger}-{\varvec{X}\left({\omega\:}_{r}\right)}^{\dagger}{\varvec{T}}_{r}^{\dagger}\left({\omega\:}_{r}\right) \right\|}_{2}^{2}$$

where † represents matrix conjugate transpose operation. We solve such an optimisation problem using an active-set al.gorithm^48^.

### Hyperspectral image retrieval of a hidden object behind the scattering media

**Fig. 3:**
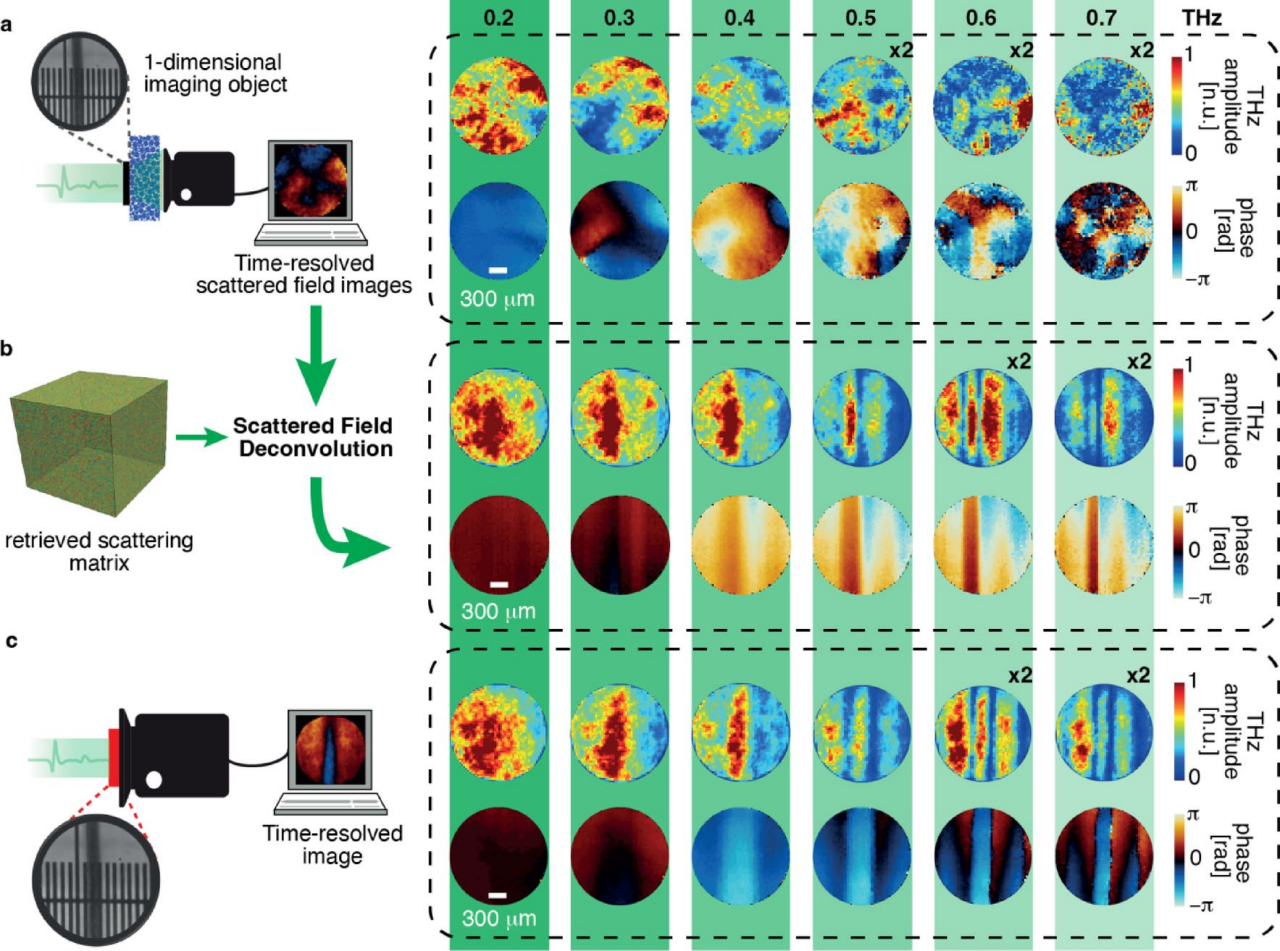
Hyperspectral image retrieval through scattering media. (**a**) Broadband scrambled field and phase profiles of a 1D imaging object (inset image: 200 $\:\mu\:m$ thick copper wire over a reference ruler with ticks spaced 100 μm) hidden behind the scattering media (video 3). (**b**) Hyperspectral image retrieval: the data in a. are deconvoluted by the reconstructed transmission matrix of a scattering sample (video 4). (**c**) Hyperspectral THz images of the 1D imaging object used in a. for comparison (video 5).

The ability to reconstruct the combinatory transfer matrix of the scatterers allows for the imaging of objects obscured by a scattering medium. Figure 3a illustrates the implementation of our image reconstruction process, where a metallic object $\:O\left(x,y\right)$ is positioned on the input surface of the scattering medium. This shapes the impinging THz field and results in convoluted spatiotemporal scattered field distribution at the output of the media as,

6 $$\:D\left({x}^{{\prime\:}},{y}^{{\prime\:}};{t}^{{\prime\:}}\right)=\iiint\:{T}_{x}\left(x,y;{x}^{{\prime\:}},{y}^{{\prime\:}};t,{t}^{{\prime\:}}\right)O\left(x,y\right){E}_{in}\left(x,y;t\right)dxdydt$$

where $\:D\left({x}^{{\prime\:}},{y}^{{\prime\:}},t\right)$ is spatiotemporal field distribution recorded by THz near field imager. To extract the information of a hidden object behind the complex media from the measurements, we conduct a standard deconvolution of the retrieved combinatory transfer matrix, which produces the time-resolved field image $\:{E}_{retreived}\left(x,y,t\right)$ ​as follows:

7 $$\:{E}_{retrieved}\left(x,y;t\right)={\mathcal{F}}^{-1}\left[\stackrel{\sim}{Tr}{\left(x,y;{x}^{{\prime\:}},{y}^{{\prime\:}};\omega\:\right)}^{-1}*\stackrel{\sim}{D}\left({x}^{{\prime\:}},{y}^{{\prime\:}};\omega\:\right)\right]$$

where $\:{\mathcal{F}}^{-1}$ and * represents the inverse time-Fourier transform and spatial convolution operation, respectively, and $\:\stackrel{\sim}{D}\left({x}^{{\prime\:}},{y}^{{\prime\:}};\omega\:\right)$ is the time-Fourier transform of the recorded signal. To perform deconvolution, the essential task involves determining the inverse of $\:\stackrel{\sim}{Tr}(x,y;{x}^{{\prime\:}},{y}^{{\prime\:}};\omega\:)$. We employed the Moore–Penrose pseudo-inversion method, implemented by a truncated singular-value decomposition. Figure 3a depicts the experimental arrangement that involves acquiring time-domain scattered fields by placing a one-dimensional image object (200 μm copper wire) at the anterior end of the scattering sample. Initially, we perform a Time-Fourier transform of the recorded signal to extract the spectroscopic information available for each pixel. As illustrated in Fig. 3a, it is evident that the transmitted field-phase profiles of the object, subsequent to exposure to a uniform THz pulse, undergo a complete scrambling process of information within the scattering sample. The scrambled information of the object is further employed with the direct deconvolution operation as delineated by Eq. (7) in order to conduct the image retrieval procedure. Intriguingly, the outcome of this reconstruction (Fig. 3b) is an image retrieval process across the extensive bandwidth of the THz spectrum. We can observe the remarkable matching with the measured direct field image without a scattering medium, in Fig. 3c. Intriguingly, because the wire is metallic, we can appreciate that the post-scattered field polarised along the wire direction, x. In the spectrum domain, the phase image of the wire is clearly accompanied by lateral echoes that represent the phase delay accumulated by the scattered field along x^49^.

## Methods

### Experimental settings and data post-processing

The experimental setting is based on a full-field THz-TDS microscope as per ref^50,51^, and shown in supplementary figure S2. A THz field generated by a large aperture LiNbO_3_ prism, excited with a tilted front pulse from a regenerative amplified source, is projected into the detection crystal (a thin LiNbO_3_) that functions as an imaging platform, as per supplementary S2. The field of view offered by THz imager is about 1.44 mm × 1.44 mm. The reflection of a large probe, impinging on the opposite side of the detection crystal performs a large-area electro-optic sampling, its relative delay being controlled by a delay stage placed on the optical THz pump. An imaging system project to orthogonal circular polarisation components of the probe beam onto a CMOS camera sensor, which results in a spatial resolution of 28.8 μm spatial period (obtained by binning the 600 × 600 image resolution output down to 50 × 50 to achieve appropriate signal-to-noise ratio). supplementary figure S3 describes the role of detection plane spatial sampling. The typical performance of the imaging setup is shown in supplementary figure S5, which serves as a benchmark for our imaging system. As we compare our results with the ongoing investigation in the optical domain, it is important to stress that the temporal coherence of scatterers is a pivotal problem at optical wavelength (i.e. the medium undergoes rapid thermal changes at scales comparable to or larger than the wavelength), this is significantly mitigated by the long THz wavelength. Hence, we did not experience any drift in the scattered pattern within the typical timeframe of an experimental session.

## Conclusion

We experimentally demonstrated a deterministic approach towards imaging through complex scattering media operating with broadband THz pulses. A large area-field detection is exploited to perform an orthogonal sampling decomposition of the scattering transmission matrix. Regardless of the relatively low density of scattering element in the medium, a near-field coupled knife-edge allows the excitation of a sufficiently large number of separable scattering modes to allow complete reconstruction of a field distribution through the medium. We performed a benchmark of this concept using a 1D image. As this approach does not exploit any statistical description of the scatterer and simply applies a super-position principle, we demonstrated full operation with broadband waveforms, which translates into the ability to reconstruct the full scattered spectrum from the object, i.e. their spectral fingerprint. This specific aspect is currently the subject of further studies. We believe our microscopy approach is a gateway to new possibilities in studying complex media, specifically scattering microstructures in biological systems.

## Electronic supplementary material

Below is the link to the electronic supplementary material.

Supplementary Material 1

Supplementary Material 2

Supplementary Material 3

Supplementary Material 4

Supplementary Material 5

Supplementary Material 6

## Data Availability

The data that support the findings of this study are openly available at https://repository.lboro.ac.uk/ at 10.17028/rd.lboro.28541129, reference number 28541129.
