# Supplementary material for: Terahertz microscopy through complex media
Source: Sci Rep. 2025 Apr 5;15:11706. doi: 10.1038/s41598-025-95951-6 (PMC11972329; doi:10.1038/s41598-025-95951-6)
Supplement: Supplementary file 1 — Supplementary Material 1 [file 41598_2025_95951_MOESM1_ESM.docx]

**Supplementary materials**

**Terahertz Ghost Imaging Through Complex Media**

Vivek Kumar^1,3^, Vittorio Cecconi^1,2^, Antonio Cutrona^1,2^, Luke Peters^1,2^, Luana Olivieri^1,2^, Juan S. Totero Gongora^1,2^, Alessia Pasquazi^1,2^, Marco Peccianti^1,2^*

^1^Emergent Photonics Lab (EPic), Department of Physics and Astronomy, University of Sussex, BN1 9QH, UK

^2^Emergent Photonics Research Centre, Department of Physics, School of Science, Loughborough University, LE11 3TU, UK

^3^Laboratoire Kastler Brossel, ENS-Universite PSL, CNRS, Sorbonne Universite, College de France, 24 rue Lhomond, 75005 Paris, France

*[m.peccianti@lboro.ac.uk](mailto:m.peccianti@lboro.ac.uk)

**The Supplementary Information comprises 6 pages and 5 figures.**

Supplementary note S1

**S1.1-Scattering sample information**

Our typical scattering sample used in the experiment is fabricated by embedding Hi-Z Silicon microparticles (150 μ𝑚 – 300 μ𝑚 from Ferroglobe) in a paraffin medium (shown in Fig S1(A)). Interestingly, the refractive index of Si (n=3.4) is largely independent of the frequency throughout the spectral range of the measurements. The embedding material, paraffin wax, is fully transparent at THz frequencies and has a specific melting point between 46-68$℃$, density of ~0.9 g/cm^3^ that offers low thermal conductivity and a high heat capacity. Hence it is practical as a host material. Hi-Z silicon does not offer significant carrier-driven absorption at the spectral range used; hence multiple scattering is solely responsible for field modulation.

Initially, we performed a series of TDS experiments to characterise scattering samples of varying thickness and scatterers mass density by measuring the transmission of coherent, single-cycle THz pulses. Further we choose scattering sample that consists of 8% fractional mass concentration for Si particles with total sample thickness of 5.53 mm.

**S2.2- Terahertz characterisation of the scattering sample**

The combined study of concepts and methodologies from the field of complex wave propagation in scattering media and THz-TDS was reported early in 2000^1,2^. Several other groups have been investigated terahertz radiation transport phenomena in ensembles of dielectric spheres^3^, subwavelength size metallic particles^4^, granular composite materials^5,6^ and random assemblies of spherical silica (SiO_2_) particles in a paraffin matrix^7^. We performed similar TDS experiments to characterise scattering samples by measuring the transmission of coherent, single-cycle THz pulses as shown in Figure S1 (B-C). The transmitted wavefronts allow extracting the mean free path, $l_{s}\left( \omega\right)$, of the sample over a broad bandwidth by measuring the transfer function of a sample^2,7^. To this end, we first collected both reference and sample waveform and obtained transfer function for a sample as,

| $H_{sample}\left( \omega\right)=\frac{E_{sample}\left( \omega\right)}{E_{ref}\left( \omega\right)}$ | (S1) |
| --- | --- |

where, $E_{sample}\left( \omega\right)$ and $E_{ref}\left( \omega\right)$ are time-Fourier transform of terahertz field profile propagated through a sample and free space, respectively. Further, scattering mean free path of a scattering sample over the entire bandwidth can be obtained as,


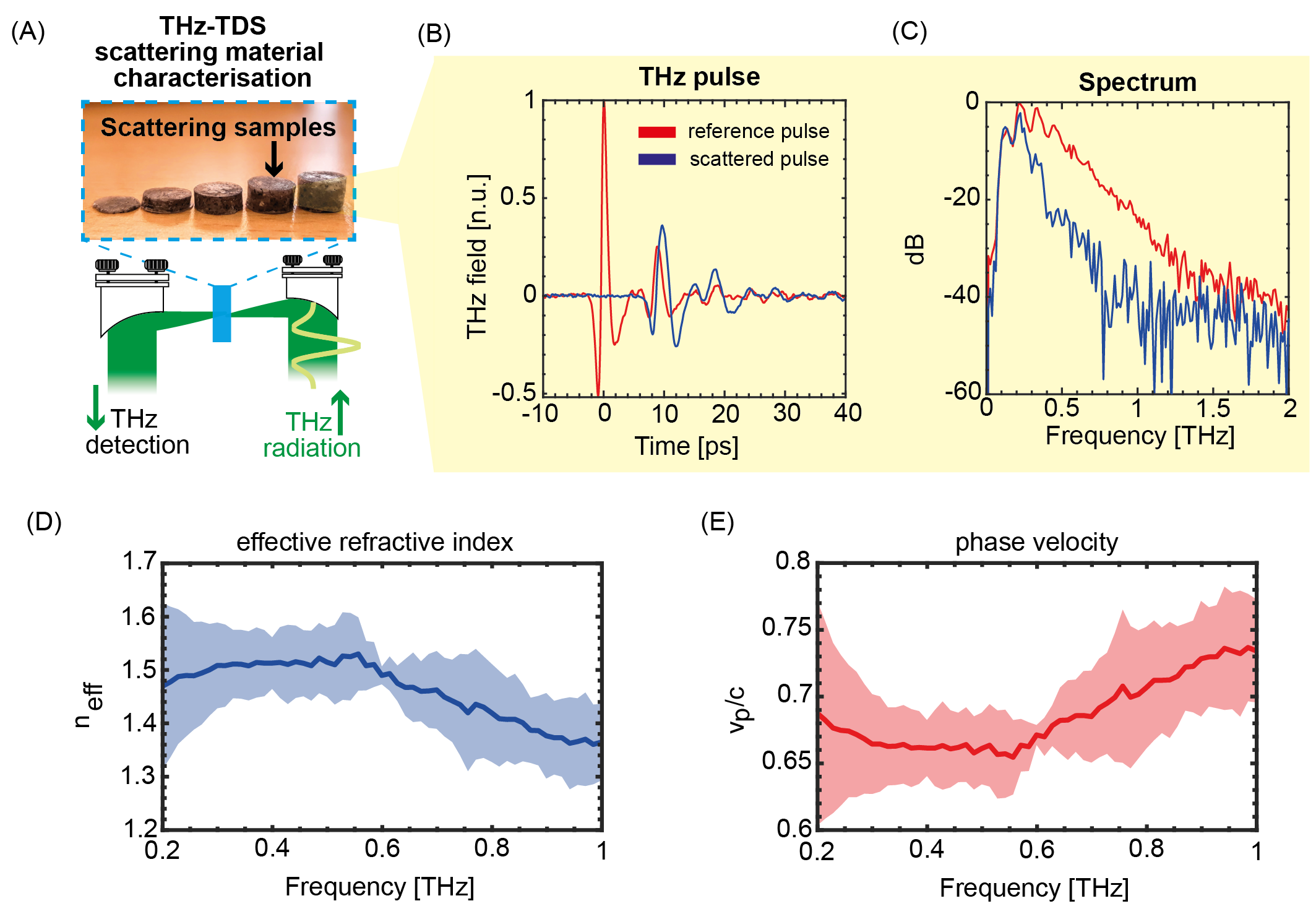


**Figure S1: Scattering sample characterisation. (A)** Schematic of THz TDS in Transmission Configuration **(B)** THz pulses for reference and sample.

| $l_{s}\left( \omega\right)=-\frac{L_{0}}{2 ln\left( H\left( \omega\right) \right)}$ | (S2) |
| --- | --- |

where, $L_{0}$ is the thickness of scattering sample.

**Supplementary note S2- Experimental setup**

The experimental setting employs a regenerative amplifier Ti: Sapphire laser system to provide an 1mJ optical pulse with a repetition rate of 1 kHz and a pulse duration of 90 fs at a central wavelength of 800 nm. As shown in Fig. S2, a beam sampler divides the laser into two beams, i.e., pump and probe. The pump beam is delayed in time with a motorised stage and undergoes front-tilt correction with the combined used of a diffraction grating (1800 lines/mm) and lenses (L1=250 mm, L2=150 mm). The pump pulse is nonlinearly converted into a THz beam via optical rectification on the Cherenkov angle of a stochiometric MgO (0.6%) prism cut Lithium Niobate crystal^8–11^. The crystal hosts an optical rectification process that exhibits maximum phase-matching also along the normal of the output facet^11^. The very large interaction length, the relatively high nonlinear susceptibility and the damage threshold of the Lithium Niobate prism allow for remarkably high optical to THz conversion efficiency (0.1% and above). Further, the THz pulse is modulated via a 10 Hz chopper obtained as an exact division of the laser repetition rate, and then it illuminates an arbitrary imaging sample. On the other hand, the probe beam is imaged on the back side of a quadratic detection crystal (a 20 𝜇𝑚 - Lithium Niobate film deposited on a glass substrate) utilising a telescopic combination of lenses L3 (300 mm), L4 (100 mm) and L5 (100 mm), L6 (100 mm). The facet at the other end of the detection crystal is coated with a highly reflective coating. The result is that the reflected probe co-propagates with the impinging THz wave, realising a large spatial area of electro-optical sampling, thanks to the THz-induced Pockels effect. Hence, the spatial distribution of the polarisation state of the reflected probe beam carriers the image of the detected THz field. Following a standard approach in practice, a large quarter wave plate, combination of lenes (L7=75 mm, L8=150 mm) and a polarising beam splitter separate the two images of the clockwise and anti-clockwise circular polarised components that are both projected onto the same CMOS camera (DMK23UP1300)^12–14^.


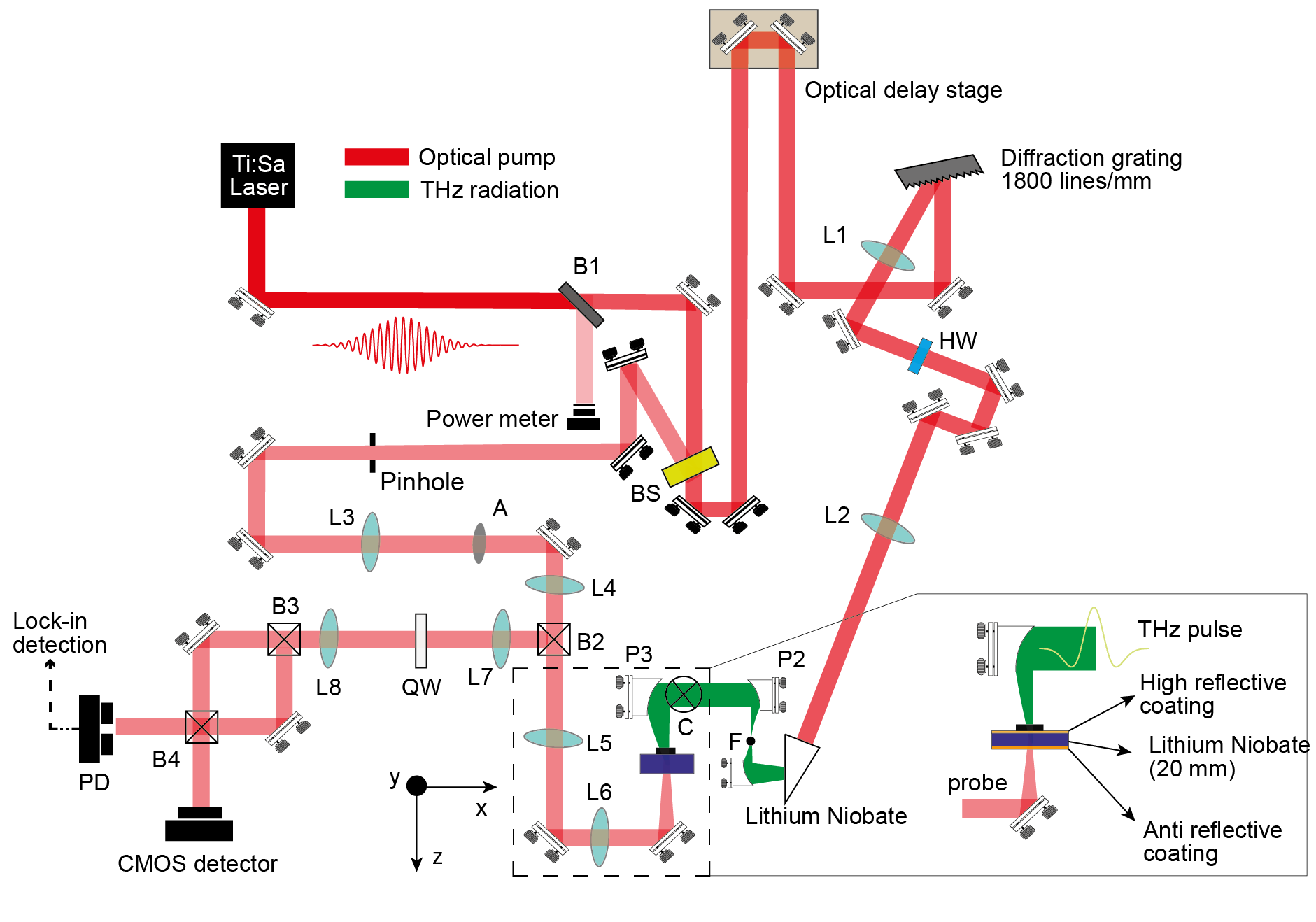


**Figure S2:** **Schematic of an experimental setup for terahertz near field imager.** B1 – 99:1 beam splitter, B2, B4 – 50:50 beam splitter, B3 – 50:50 polarised beam splitter, BS – Beam sampler, C – chopper, HW – half waveplate, PD – balanced photodiodes, A – attenuator, F – filter, QW – quarter waveplate, L(1-8) – plano-convex lens (L1=250 mm, L2=150 mm, L3=300 mm, L4=100 mm, L5=100 mm, L6=100 mm, L7=75 mm, L8=150 mm), P – Parabolic mirrors (P1=25.4 mm, P2=76.2 mm, P3=50.3 mm).

**Supplementary note S3- The role of detection plane spatial sampling**

A fundamental aspect in assessing the scattered field is related to the broadening of the output spatial spectrum induced by scattering. This is particularly relevant when we consider a near-field detection, where transverse spatial-frequency components can have sub-wavelength periods, i.e. momentum exceeding the vacuum propagation constant. From this argument, it descends that the output spatial sampling period during the image acquisition (in the two transverse dimensions) should be significantly smaller than the wavelength. Besides, the CMOS array sampling period ($4.8 \mu m)$results in the resolving power of the imaging system (better than $7 \mu m$ ) orders of magnitude smaller than the peak terahertz wavelength and largely within the optical diffraction limit of the probe assembly. Conversely, large detection areas correspond to higher detected signals and lower signal-to-noise ratio. Figure S3(A) and S3(B) present the typical benefit obtained by binning together the camera element to obtain a macro-pixel of a given size. In terms of procedure, then, the output sampling will be chosen, analysing the output spatial spectrum at different scales, determining ∆C from the roll-off of the spatial spectrum at high frequency, and keeping the spatial sampling $\Delta_{x}$ one order of magnitude smaller than ∆C (corresponds to a Nyquist frequency Limit of 5/∆C).


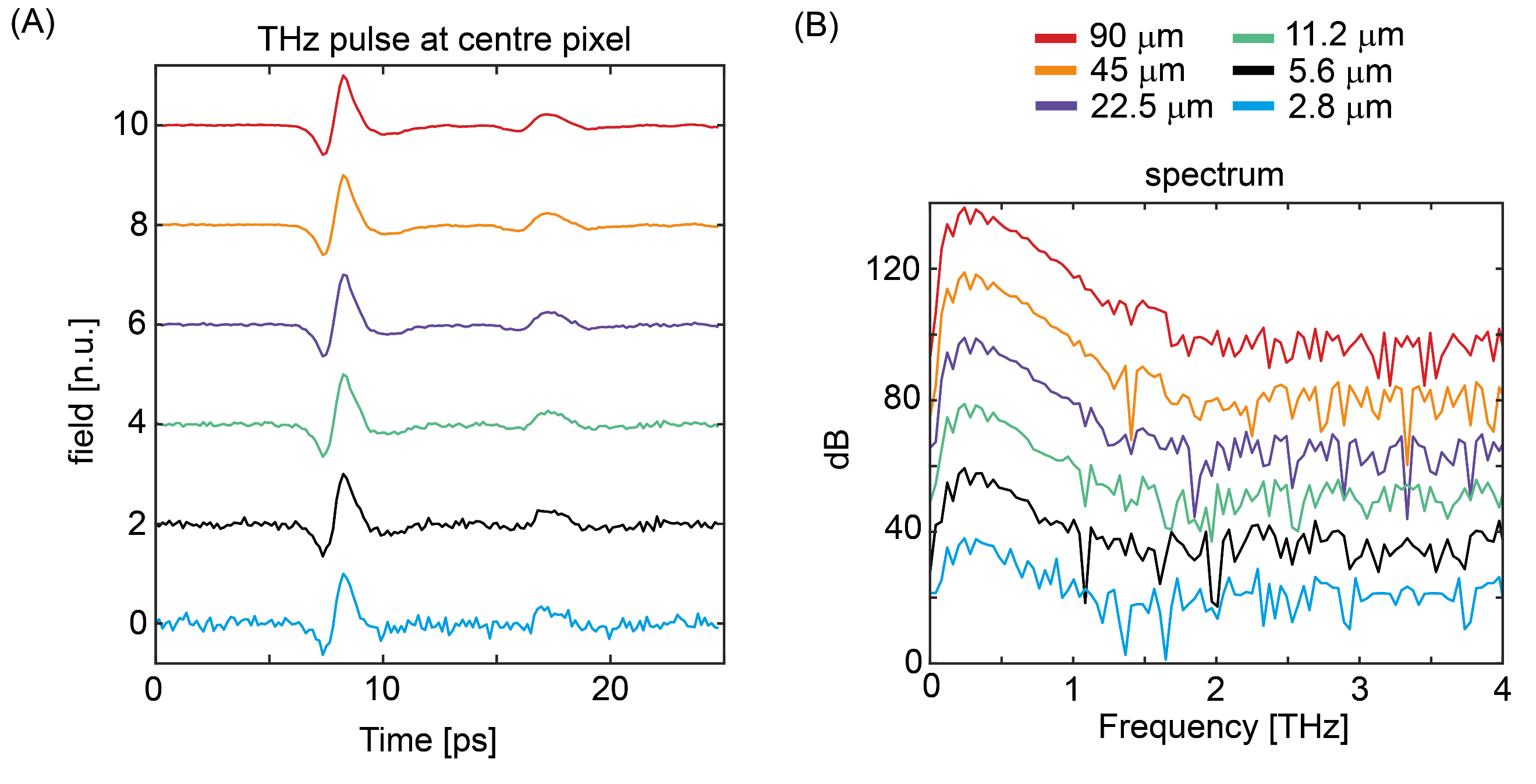


**Figure S3: Spatial sampling of detection plane. (A-B)** Signal-to-noise variation in THz pulse and its spectrum collected at a centre macro pixel by varying pixel size.

**Supplementary figures**


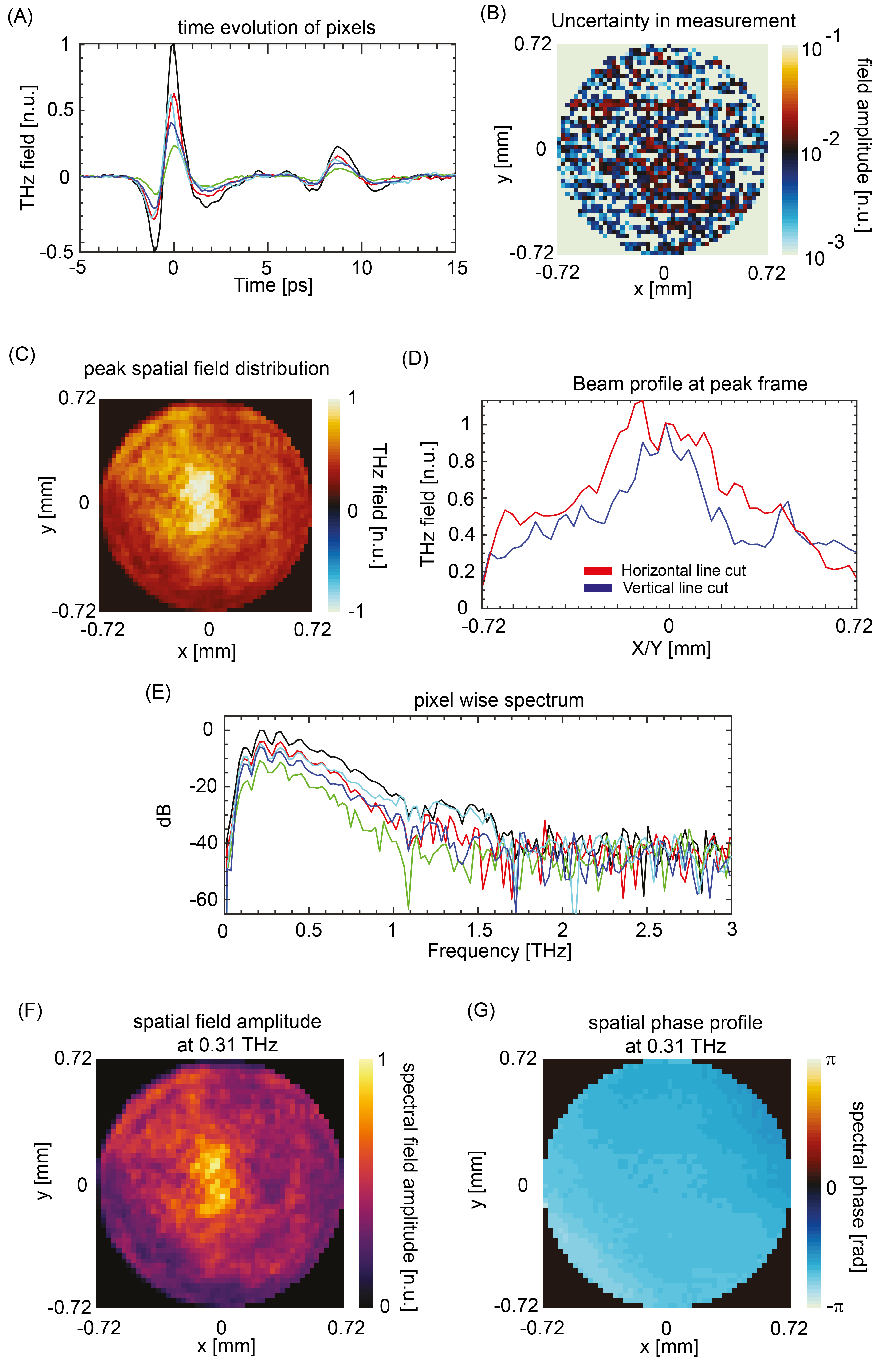


**Figure S4:** **Time-resolved reference beam profile obtained from terahertz near-field imager. (A)** Time-evolution of some exemplary pixels. **(B)** Uncertainty (noise) map in spatial field measurements. **(C)** Spatial field distribution at 0 ps. **(D)** Beam profile obtained for spatial field distribution at the peak of pulse. **(E)** Terahertz reference spectrum corresponding to various pixels. **(F)** Spatial field amplitude profile at 0.31 THz. **(G)** Spatial phase distribution at 0.31 THz.


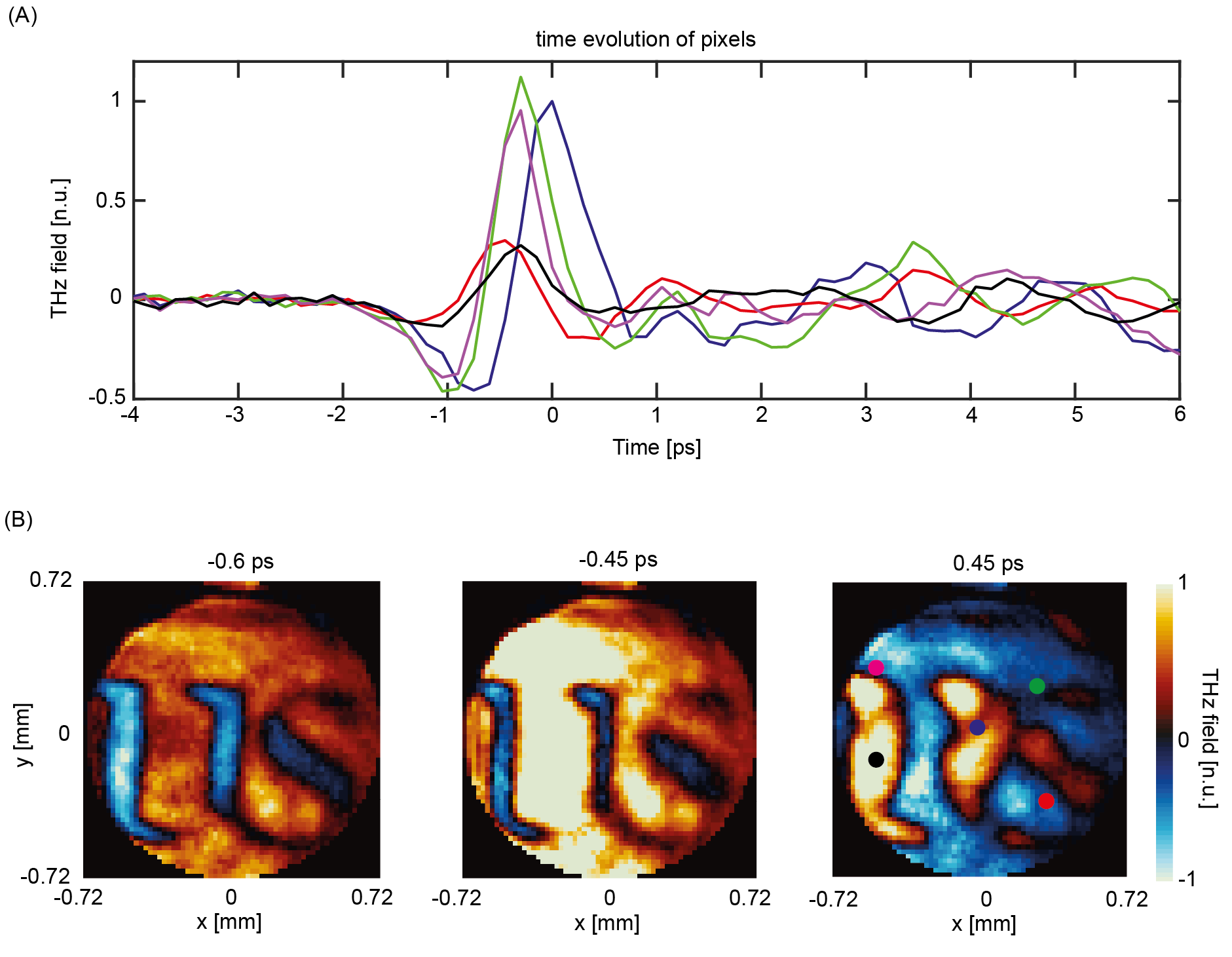


**Figure S5:** **Time-resolved image of metallic mask collected by terahertz near field imager.** (A) Time evolution of various pixels. (B) Spatial field distribution of metallic mask at -0.6 ps, -0.45 ps and 0.45 ps.

**Supplementary Video S1:** Time evolution of spatial field distribution transmitted through scattering media.

**Supplementary Video S2:** Spatial field and phase distributions of the transmitted field over the board THz spectrum.

**Supplementary Video S3:** Broadband scrambled field and phase profiles of a 1D imaging object hidden behind the scattering media.

**Supplementary Video S4:** Hyperspectral image retrieval using reconstructed transmission matrix of a scattering sample.

**Supplementary Video S5:** Hyperspectral THz images of the 1D imaging object without scattering media.

**References**

(1) Pearce, J.; Mittleman, D. M. Propagation of Single-Cycle Terahertz Pulses in Random Media. *Opt. Lett., OL* **2001**, *26* (24), 2002–2004. https://doi.org/10.1364/OL.26.002002.

(2) Pearce, J.; Mittleman, D. M. Scale Model Experimentation: Using Terahertz Pulses to Study Light Scattering. *Phys. Med. Biol.* **2002**, *47* (21), 3823–3830. https://doi.org/10.1088/0031-9155/47/21/321.

(3) Cheville, R. A.; McGowan, R. W.; Grischkowsky, D. Time Resolved Measurements Which Isolate the Mechanisms Responsible for Terahertz Glory Scattering from Dielectric Spheres. *Phys. Rev. Lett.* **1998**, *80* (2), 269–272. https://doi.org/10.1103/PhysRevLett.80.269.

(4) Chau, K. J.; Elezzabi, A. Y. Terahertz Transmission through Ensembles of Subwavelength-Size Metallic Particles. *Phys. Rev. B* **2005**, *72* (7), 075110. https://doi.org/10.1103/PhysRevB.72.075110.

(5) Kaushik, M.; Ng, B. W.-H.; Fischer, B. M.; Abbott, D. Terahertz Scattering by Granular Composite Materials: An Effective Medium Theory. *Appl. Phys. Lett.* **2012**, *100* (1), 011107. https://doi.org/10.1063/1.3674289.

(6) Born, P.; Holldack, K. Analysis of Granular Packing Structure by Scattering of THz Radiation. *Review of Scientific Instruments* **2017**, *88* (5), 051802. https://doi.org/10.1063/1.4983045.

(7) Gentilini, S.; Missori, M.; Ghofraniha, N.; Conti, C. Terahertz Radiation Transport in Photonic Glasses. *Annalen der Physik* **2020**, *532* (8), 2000005. https://doi.org/10.1002/andp.202000005.

(8) Stepanov, A. G.; Hebling, J.; Kuhl, J. Efficient Generation of Subpicosecond Terahertz Radiation by Phase-Matched Optical Rectification Using Ultrashort Laser Pulses with Tilted Pulse Fronts. *Appl. Phys. Lett.* **2003**, *83* (15), 3000–3002. https://doi.org/10.1063/1.1617371.

(9) Hebling, J.; Stepanov, A. G.; Almasi, G.; Bartal, B.; Kuhl, J. Tunable THz Pulse Generation by Optical Rectification of Ultrashort Laser Pulses with Tilted Pulse Fronts. *Applied Physics B: Lasers and Optics* **2004**, *78* (5), 593–599. https://doi.org/10.1007/s00340-004-1469-7.

(10) Hebling, J.; Yeh, K.-L.; Hoffmann, M. C.; Bartal, B.; Nelson, K. A. Generation of High-Power Terahertz Pulses by Tilted-Pulse-Front Excitation and Their Application Possibilities. *JOSA B* **2008**, *25* (7), B6–B19.

(11) Hirori, H.; Doi, A.; Blanchard, F.; Tanaka, K. Single-Cycle Terahertz Pulses with Amplitudes Exceeding 1 MV/Cm Generated by Optical Rectification in LiNbO[Sub 3]. *Applied Physics Letters* **2011**, *98* (9), 091106. https://doi.org/10.1063/1.3560062.

(12) Blanchard, F.; Nkeck, J. E.; Matte, D.; Nechache, R.; Cooke, D. G. A Low-Cost Terahertz Camera. *Applied Sciences* **2019**, *9* (12), 2531. https://doi.org/10.3390/app9122531.

(13) Blanchard, F.; Doi, A.; Tanaka, T.; Tanaka, K. Real-Time, Subwavelength Terahertz Imaging. *Annu. Rev. Mater. Res.* **2013**, *43* (1), 237–259. https://doi.org/10.1146/annurev-matsci-071312-121656.

(14) Blanchard, F.; Tanaka, K. Improving Time and Space Resolution in Electro-Optic Sampling for near-Field Terahertz Imaging. *Opt. Lett., OL* **2016**, *41* (20), 4645–4648. https://doi.org/10.1364/OL.41.004645.
